# Phenotype and genotype in a Taiwanese girl with Sotos Syndrome

**DOI:** 10.37796/2211-8039.1372

**Published:** 2022-09-01

**Authors:** Wei-De Lin, Chung-Hsing Wang, Fuu-Jen Tsai, I-Ching Chou

**Affiliations:** a Department of Medical Research, China Medical University Hospital, Taichung, Taiwan; b School of Post Baccalaureate Chinese Medicine, China Medical University, Taichung, Taiwan; c Division of Genetics and Metabolism, Children’s Hospital of China Medical University, Taichung, Taiwan; d School of Medicine, China Medical University, Taichung, Taiwan; e Department of Medical Genetics, China Medical University Hospital, Taichung, Taiwan; f School of Chinese Medicine, China Medical University, Taichung, Taiwan; g Department of Medical Laboratory Science and Biotechnology, Asia University, Taichung, Taiwan; h Division of Pediatrics Neurology, China Medical University, Children’s Hospital, Taichung, Taiwan; i Graduate Institute of Integrated Medicine, China Medical University, Taichung, Taiwan

**Keywords:** Microdeletion, Psychological developmental delay, Sotos syndrome

## Abstract

Rare copy number variations have been linked to an important source of mutation in many psychopathological traits and neurodevelopmental disorders. In this study, we describe a Taiwanese girl with mental retardation and mild macrocephaly who underwent a childhood psychological evaluation for several years first. When she was 5 years old, she came to our hospital for further diagnosis. We conducted molecular cytogenetic tests and confirmed she actually has Sotos syndrome. We compared our case with two others with very similar deletion regions, but their phenotypes were heterogeneous. Sotos syndrome is very rare in Taiwan, and it is suggested that genetic analysis should be considered early if symptoms of this case are observed.

## 1. Introduction

Copy number variations (CNVs) are polymorphisms in the number of copies of chromosomal microdeletions and microduplications (genomic rearrangements). They range from 1 kb to several Mb and have been reported to be causal suspects in various developmental disorders. CNVs may change the dosage balance of allele genes, generate new recombination of exons between different genes, or disrupt regulatory regions, resulting in variations in gene expression or altered protein structure and activity. Therefore, if CNVs are large and affect multiple genes, they are likely to be involved in different phenotypes, including disease sensitivity. CNVs can be inherited from a parent or arise *de novo* in an individual. Usually, *de novo* CNVs are more likely to have damaging effects [1].

Rare CNVs have been linked to an important source of mutation in many psychopathological traits and neurodevelopmental disorders, such as intellectual disability, autism spectrum disorders, attention-deficit hyperactivity disorder, epilepsy, and schizophrenia [2]. Array comparative genomic hybridization (aCGH) is a high-resolution technology to detect rare CNVs on chromosomes that cannot be observed using conventional cytogenetic karyotyping [2]. Herein, we report on a 5-year-old Taiwanese girl with a phenotype of psychological developmental delay and mild macrocephaly with a high broad forehead and identified a *de novo* 1.86-Mb microdeletion at 5q35.2-q35.3 associated with Sotos syndrome.

## 2. Case and methods

The index patient was the first child of healthy non-consanguineous Taiwanese parents and was born by cesarean section at 38 weeks of gestational age following an uneventful pregnancy/delivery, except for amniotic fluid inhalation. The ages of the parents at conception were 32 (father) and 27 (mother) years. The birth weight was 3500 g (50–85th percentile), length of 53 cm (97th percentile), and head circumference was 38 cm (above the 97th percentile). Due to the large head circumference, electroencephalography and brain computerized tomography were performed shortly after birth and the results were unremarkable.

The parents reported that she started climbing at the age of 12 months and walked without support at 24 months. When she was 15 months old, she began receiving physical, language, and functional therapies. She was able to say “mama” and “papa” at 36 months and repeat the words overhead in conversation at the age of 5 years old. However, there has been no full sentence development yet. At the age of 34 months, the evaluation of psychomotor development according to the Bayley Scales of Infant and Toddler Development (BSID-III) was performed and showed that her cognition fell into the range of slow development (BSIDIII: CCI = 65; 1 percentile), with a developmental age of approximately 17 months old; language skills fell under the scope of developmental delay (BSID-III: Lang = 65; 1 percentile), with approximately 14 months of expression and 13 months of understanding.

The patient started kindergarten at 5 years of age. She could express her physical needs, such as going to the toilet and hunger using voice or simple words. She was easily attracted to things that interested her, such as mobile phones and e-books. Due to her lack of language ability, she often expressed herself as a means of pushing or beating other people. For the continuation of the psychotherapeutic interview and supportive psychotherapy, she was evaluated using the Adaptive Behavior Assessment System, Second Edition (ABAS-II) at 65 months of age. Her language and communication development was 16–26 months old, social and personality development was 21–40 months old, and perception and cognitive development were 11–29 months old. All these developmental parameters fell into the scope of developmental delay. The scores of the General Adaptive Composite were 70 (percentile rank [PR] = 2; 95% CI = 68–72), which fell within the critical range. The scores for the three subdomains were: 60 for conceptual (PR = 0.4), 93 for social (PR = 32), and 61 for practical (PR = 0.5).

**Fig. 1:**
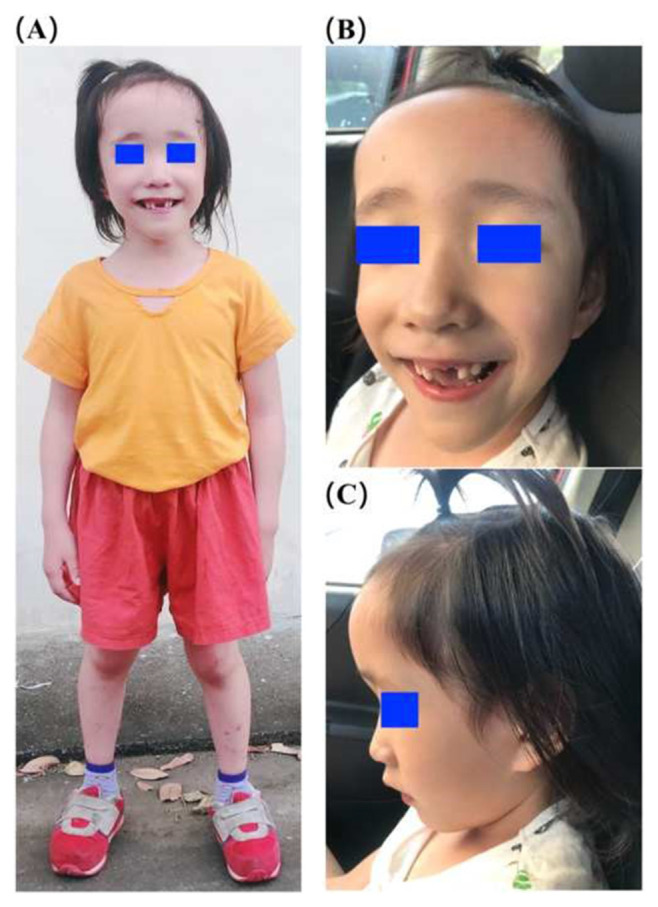
Clinical photographs of the patient at the age of 60 months. (A) Full-body frontal photograph. (B, C) Frontal and side photos of the head show the long face, sparse hair in the frontoparietal area, sparse eyebrows, hypertelorism, malar hypoplasia, thin philtrum, mild depressed and broad nasal root, prominent forehead, normal lips, and normal tongue.

The patient was brought to the genetic outpatient clinic of our hospital at the age of 5 years (62 months) for genetic counseling regarding the psychological developmental delay. Her body height, weight and body mass index were 112.7 cm (50–85th percentile), 20.8 kg (85th percentile), and 16.3 kg/m^2^ (50–85th percentile), respectively, according to the New Growth Charts for Taiwanese Children and Adolescents [3]. The Tanner stage was B1P1. On physical examination, she had a long face with a broad forehead, sparse eyebrows, mildly depressed nasal root, large ears, happy and friendly characteristics, above the mean stature, and macrocephaly (head circumference 57 cm, above the 97th percentile) [Fig. 1 A,B,C].

Array-CGH was performed on the patient’s DNA extracted from peripheral blood using SurePrint G3 Human CGH ISCA (Agilent Technologies, Santa Clara, CA, USA), and data were analyzed using the Agilent CytoGenomics (v. 4.0.3.12) software.

## 3. Results and discussion

**Fig. 2:**
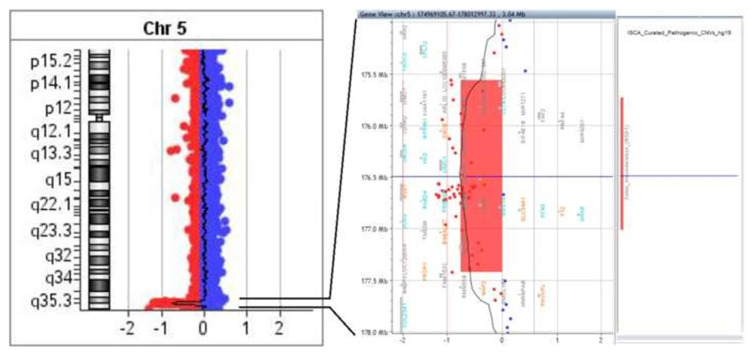
Array-CGH chromosome 5 showing the deleted segment in chromosome 5q35.2-q35.3 in our patient, size of deletion 1.86 Mb, the deleted genes were shown within the deleted region.

According to Array-CGH result, we identified a 1.863 Mb deletion of 5q35.2–35.3 (arr[GRCh37](1-22,X)x2, 5q35.2q35.3 (175559343_177422760)x1), which encompasses the NSD1 gene and other 49 genes and transcripts [Fig. 2, Supplementary Table 1]. This result is compatible with the diagnosis of Sotos syndrome.

Sotos syndrome (OMIM 117550) is a disease characterized by overgrowth and intellectual disability with an incidence of approximately 1 in 15,000 live births [4]. Its inheritance pattern is autosomal dominant and is caused by a deletion or mutation in the *NSD1* gene, which is mapped to 5q35.2-q35.3 [4,5]. The proportions of intragenic and microdeletion mutations in Sotos syndrome differ between countries. In East Asia, studies from Japan reported that 52% of Japanese patients with Sotos syndrome were microdeletions, but only 10–15% were observed in non-Japanese ethnicities [6,7]. In 2005, it was reported that in Hong Kong, Southern China, only 12% of Sotos syndrome cases were the microdeletion type, similar to that in a Western study [8]. In a study in Korea, 53% of Korean patients had 5q35 microdeletion [9]. In Taiwan, Sotos syndrome was very rare, and only one prenatal diagnosis case was reported; it was a girl with 5q35.2-q35.3 microdeletion but without follow-up information [10]. In this study, we describe the case of a patient with developmental delay who underwent a childhood mental evaluation for several years and confirmed the diagnosis of Sotos syndrome using molecular cytogenetic methods in the final.

Although Sotos syndrome cases are rare, we estimated that the microdeletion type of mutation should be dominant in Taiwanese Sotos syndrome.

The *NSD1* gene encodes the nuclear receptor set domain protein 1, which is a histone methyl-transferase that methylates lysine residues on histone tails [11]. Histones are structural proteins that bind to DNA, and methylation of histones is often associated with the transcription regulation of genes. *NSD1* gene mutations disrupt the normal regulation of genes involved in cell/tissue growth and development [12].

The deleted region contains 50 genes and transcripts [Supplementary Table 1]; besides *NSD1*, six genes, including *GPRIN1*, *SNCB*, *UNC5A*, *ZNF346*, *RGS14*, and *DBN1*, are also highly associated with brain function, neural system development, neuron protecting or a nonprogressive cerebral disorder with mental retardation [13–20]. The other two genes, *RNF44* and *ARL10*, are highly expressed in brain, although their function is unclear [21,22]. In general, individuals with microdeletion of *NSD1* and neighboring genes in the 5q35 region present with less severe tall stature and more severe intellectual disability, compared to that in individuals with intragenic mutations [23].

According to the literature reviewed, two cases had deletion regions very similar to the case that we described here. One was a 3.5-year-old African-American girl, and the other was a 2.5-year-old African boy [24,25]. The clinical features were compared and summarized in Table 1. All of them had over growth, either at birth or at the time of reporting. They had long face, prominent and high forehead, sparse eyebrow, depressed nasal root, large ears, short philtrum, pointy chin, and macrocephaly. In neurological and psychiatric symptoms, all of them were development delay in speech and communication, social and personality, perception and cognitive. Considering these symptoms and signs, several disease should be suspected, such as Leukodystrophies, neurocutaneous syndromes (phakomatosis), lysosomal storage disorders and Noonan syndrome. Genetic testing can help clarify the cause of the disease. The deletion information was converted to the same database set (GRCh38/hg38).

The deletion region in our case: Chromosome 5:176132340-177995759 (1.86 Mb), AfricanAmerican girl: Chromosome 5: 176050165-177680777 (1.63 Mb), African boy: Chromosome 5: 176006844-178003495 (2Mb). The deletion region of the African boy was the largest in these three cases; however, the African-American girl had more severe dysmorphisms, including cleft lip palate and patent ductus arteriosus (PDA), which were not found in the other two cases. Cleft lip palate was not reported in any Sotos syndrome except in this African-American girl; PDA was observed in 19% of microdeletion mutation type Sotos syndrome cases [5]. The number of gene and transcript deletions in the African boy was 52, 47 in the African-American girl, and 50 in our case. Most of these gene and transcript functions were unknown [Supplementary Table 1]. Although the deletion regions were very similar, the phenotypes were heterogeneous. More studies are needed to elucidate the functions and interactions of genes located in this region.

In our patient, some peculiar characteristic facial appearance was observed, including macrocephaly since birth, prominent forehead, high anterior hairline, pointy chin, and subtle down-slanting palpebral fissures, all of which are compatible with the diagnosis of Sotos syndrome. Other physical symptoms that are commonly described in Sotos syndrome’ cases, such as seizures, scoliosis, cardiac anomalies, renal anomalies, and joint laxity, were not observed in this patient at this time, but we will closely monitor for these at future follow-ups.

In conclusion, microdeletion or duplication is one of the most important causes of physical and mental developmental disorders. Although our case is generally compatible with typical Sotos syndrome, after detailed array-CGH analysis and comparison with other similar deletion cases, we can extend more data on genotype and phenotype correlation. In addition, Sotos syndrome is rare in Taiwan, and it is suggested that genetic analysis (array-CGH, target gene-*NSD1* sequencing) should be considered early if symptoms of this case are observed. Genetic diagnostic results can provide more information for the patients’ future development and genetic counseling for the family.

## Supplementary Data

**Supplementary Table-S1:** Genes and transcripts list deletion in our case and the other two similar reported cases.

Gene	Location (GRCh38)	Related OMIM disease	Inheritance	Note #
AC139491.1	5:176049677-176062021	–		
*FAM153B*	5:176084962-176114798	–		
AC139493.1	5:176203313-176203710	–		
*SIMC1*	5:176238423-176345989	n/a	n/a	
*KIAA1191*	5:176346061-176361764	–		
AC138956.1	5:176347940-176353584	–		
*ARL10*	5:176365486-176381909	–		highly expressed in the brain
*NOP16*	5:176383945-176388762	n/a	n/a	nucleolar protein
*HIGD2A*	5:176388750-176389761	–		a subunit of the cytochrome c oxidase complex (complex IV)
*CLTB*	5:176392500-176416539	n/a	n/a	encodes one of two clathrin light chain proteins which are believed to function as regulatory
AC010297.1	5:176439089-176439238	–		
*FAF2*	5:176448384-176510074	n/a	n/a	highly expressed in peripheral blood of patients with atopic dermatitis
*RNF44*	5:176526711-176537402	n/a	n/a	expression in adult and fetal human brain regions
*CDHR2*	5:176542510-176595974	–		
*GPRIN1*	5:176595801-176610156	n/a	n/a	wide distribution in human brain tissue and the central nervous system with highest expression in the spinal cord
*SNCB*	5:176620081-176630534	Dementia with Lewy body	AD	Be concentrated in presynaptic nerve terminals; increased Akt signaling activity and were resistant to neurotoxic effects of the pesticide rotenone
MIR4281	5:176629438-176629500	–		micro RNA
*EIF4E1B*	5:176630617-176646595	–		
*TSPAN17*	5:176647483-176659051	–		
AC113391.1	5:176707355-176726243	–		
LINC01574	5:176743204-176743871	–		long intergenic non-protein coding RNA
*UNC5A*	5:176810518-176880898	n/a	n/a	developing mouse revealed highest Unc5a expression in central nervous system
*HK3*	5:176880868-176899346	n/a	n/a	Hexokinases 3
*UIMC1*	5:176905005-177006779	n/a	n/a	
*ZNF346*	5:177022695-177067985	n/a	n/a	nucleolar protein, cell growth and survival, protecting neurons
*FGFR4*	5:177086914-177098144	n/a	n/a	a tyrosine kinase and cell surface receptor for fibroblast growth factors; involved in several pathways: cell proliferation, differentiation, migration, lipid metabolism, bile acid biosynthesis, vitamin D metabolism, glucose uptake, and phosphate homeostasis
*NSD1*	5:177133772-177300213	Sotos syndrome 1	AD	
*RAB24*	5:177301197-177303719	n/a	n/a	Ras-associated protein 24
*PRELID1*	5:177303798-177306949	n/a	n/a	
*MXD3*	5:177307434-177312757	n/a	n/a	
*LMAN2*	5:177331566-177351668	n/a	n/a	binds high mannose type glycoproteins and may facilitate their sorting, trafficking and quality control
AC146507.1	5:177346072-177346426	–		
*RGS14*	5:177357923-177372596	n/a	n/a	transcripts at high levels in brain, spleen, and lung
*SLC34A1*	5:177384433-177398848	Fanconi renotubular syndrome 2	AR	a major part in phosphate homeostasis
		Hypercalcemia, infantile, 2	AR	a major regulator of Pi homeostasis and is
		Nephrolithiasis/osteoporosis, hypophosphatemic, 1	AD	necessary for normal skeletal development
*PFN3*	5:177400108-177400661	n/a	n/a	
*F12*	5:177402140-177409564	Angioedema, hereditary, type III	AD	recurrent skin swelling, abdominal pain
		Factor XII deficiency	AR	attacks, and episodes of upper airway obstruction; coagulation cascade
*GRK6*	5:177426793-177442901	n/a	n/a	G protein-coupled receptor kinase 6
*PRR7*	5:177446813-177456286	n/a	n/a	
*DBN1*	5:177456935-177473637	n/a	n/a	neurite outgrowth, brain maturation; Drebrin (125 kD) expression was significantly decreased in the temporal and frontal cortex of both Down syndrome and Alzheimer disease patients, underlie cognitive dysfunction.
*PDLIM7*	5:177483393-177497604	n/a	n/a	These proteins are involved in cytoskeleton
AC145098.1	5:177494994-177503647	–		
*DOK3*	5:177503783-177510382	n/a	n/a	
*DDX41*	5:177511576-177516961	Familial myeloproliferative/lymphoproliferative neoplasms, adult-onset	AD	interacted with several spliceosomal proteins, tumor suppressor gene
*FAM193B*	5:177519788-177554563	n/a	n/a	
*TMED9*	5:177592202-177597242	–		
*B4GALT7*	5:177600131-177610330	Ehlers-Danlos syndrome, spondylodysplastic type, 1	AR	involved in the synthesis of the glycosaminoglycan-protein linkage in proteoglycans
AC139795.1	5:177626435-177672149	–		
AC138819.1	5:177682293-177713969	–		
*FAM153A*	5:177723364-177753206	–		
AC140125.1	5:177801203-177803344	–		
AC106795.1	5:177809480-177882383	–		
*PROP1*	5:177992234-177996242	Pituitary hormone deficiency, combined, 2	AR	a crucial role in the proper development of somatotrophs, lactotrophs, thyrotrophs, and gonadotrophs

AD, autosomal dominant; AR, autosomal recessive; n/a, not available; -, no record in OMIM.

# https://www.ncbi.nlm.nih.gov/gene/

## Figures and Tables

**Table 1:** Summary of clinical characteristics of three cases of microdeletion 5q35.2-q35.3.

	Case 1	Case 2	Case 3
			
	This study	Mubungu et al., 2020	Peredo et al., 2013
Ethnicity	Taiwan Han population	African	African American
		Congo	USA
Sex	Female	Male	Female
Age (reported)	5-year-old	2.5-year-old	3.5-year-old
Deletion region	hg19: 5q35.2-q35.3	hg19: 5q35.2-q35.3	hg18: 5q35.2-q35.3
	175559343-177422760	175433847-177430496	175409774-177040384
Convert to GRCh38/hg38	176132340-177995759	176006844-178003495	176050165-177680777
Deletion size	1.86 Mb	2 Mb	1.631 MB
Birth Height (cm)	53 (97th percentile)	53 (85th percentile)	50.8 (50–75th percentile)
Weight (kg)	3.5 (50–85th percentile)	3.89 (74th percentile)	3.26 (50th percentile)
Head circumference (cm)	38 (>97th percentile)	36.5 (63rd percentile)	38 (>95th percentile)
Reported Height (cm)	112.7 (50–85th percentile)	101 (98th percentile)	101.9 (90th percentile)
Weight (kg)	20.8 (85th percentile)	20 (>99th percentile)	20.6 (>95th percentile)
Head circumference (cm)	57 (>97th percentile)	53.5 (>99th percentile)	n/a
Outward characteristics	Long face with broad forehead, sparse eyebrows, mild depressed nasal root, large ears, above the mean stature, and macrocephaly	Long face, prominent forehead, sparse eyebrows, malar hypoplasia and reddish, depressed nasal root, short and deep philtrum, thick lip, macroglossia, large ears, short fingers, partial syndactyly of toes	Cleft lip and palate, prominent and high forehead, hypertelorism, hypoplasia of the middle face with depressed nose, prominent chin
Neurological and psychiatric symptoms	happy and friendly characteristics, developmental delay of language and communication, social and personality, perception and cognitive	Speech development delay, moderate impairment of cognitive, communicative, motor, and socialemotional performances	Speech delay, mild cerebral palsy, global developmental delay
Heart, vascular, and spine	No finding	Normal	Patent ductus arteriosus
Other clinical symptoms	–	–	Asthma

n/a, not available.
